# Development and Implementation of Societal Influences Survey Questionnaire (SISQ) for Peoples during COVID-19 Pandemic: A Validity and Reliability Analysis

**DOI:** 10.3390/ijerph17176246

**Published:** 2020-08-27

**Authors:** Dian-Jeng Li, Wei-Tsung Kao, Vincent Shieh, Frank Huang-Chih Chou, Huei-Wen Angela Lo

**Affiliations:** 1 Kaohsiung Municipal Kai-Syuan Psychiatric Hospital, Kaohsiung 80708, Taiwan; 030854@gmail.com (W.-T.K.); f50911.tw@yahoo.com.tw (F.H.-C.C.); 2 Department of Nursing, Meiho University, Pingtung 91200, Taiwan; 3 Graduate Institute of Gender Education, National Kaohsiung Normal University, Kaohsiung 80201, Taiwan; vshieh123@gmail.com; 4 Faculty of Medicine, College of Medicine, Kaohsiung Medical University, Kaohsiung 80708, Taiwan

**Keywords:** COVID-19, social influences, mental health, validity, reliability

## Abstract

The emergence of Coronavirus disease 2019 (COVID-19) had rapidly spread since FEB/MAR 2020. Policy to prevent transmission of COVDI-19 resulted in multi-dimensional impact on social interaction. We aimed to develop a beneficial survey tool with favorable quality and availability, the Societal Influences Survey Questionnaire (SISQ), to evaluate social influences on people during this pandemic. The SISQ was developed with 15 items and 4-point Likert scales consisting of five factors. These include social distance, social anxiety, social desirability, social information, and social adaptation. Construct validity and reliability were performed to verify the SISQ. A total of 1912 Taiwanese were recruited. The results demonstrated that the SISQ has acceptable reliability, with Cronbach’s alphas ranging between 0.57 and 0.76. The SISQ accounted for 58.86% and satisfied the requirement of Kaiser–Mayer–Olkinvalues (0.78) and significant Bartlett’s Test of sphericity. Moreover, the confirmatory factor analysis fit indices also indicated the adequacy of the model. As for multiple comparison, females scored higher than males in factor of social distance. Unemployed participants and those without partners scored higher in several domains of factors. The survey method and survey instrument prove reliable and valuable, also providing different categories of assessment results regarding social influences and their impacts. Further studies are warranted to extend the applicability of SISQ.

## 1. Introduction

### 1.1. COVID-19, A 21st-Century Epidemic

The emergence of a new virus, Coronavirus (COVID-19), emerged at the end of 2019. It rapidly spread [1], becoming an official global pandemic by 11 March 2020 according to the World Health Organization [2]. Over 740,000 deaths were confirmed by August 2020. Although most of the infected patients developed mild symptoms, such as dry cough and fever, some developed critical and fatal complications [3]. This has led to extreme public concern. The pandemic strongly affects daily lives, resulting in a heavy burden on social welfare and healthcare systems [1]. Furthermore, the unemployment rate due to infection control measures and economic decline has resulted in a massive public crisis [4,5]. The impact of COVID-19 is multi-dimensional. It has an immense effect on daily lives. Two epidemiological studies demonstrated the massive psychological impacts on Saudi and Bangladesh populations [6,7]. In addition, massive impact on social activities should not be neglected.

### 1.2. Massive Impact of COVID-19 and Previous Epidemics on Social Activities

Infection control policies for epidemics, such as social distancing, significantly changes peoples’ social activities and lifestyles. Social distancing of 5 feet indoors and 3.3 feet outdoors during the COVID-19 pandemic is beneficial to everyone, especially those with hypertension, cardiovascular disease, diabetes and several other chronic diseases [8]. However, the interference on social activities may have substantial mental health impacts [9]. A comparison to historic epidemics/pandemics may help us understand what to expect during the current COVID-19 pandemic and allow us to craft better response and coping measures. Previous epidemics, such as Severe Acute Respiratory Syndrome (SARS), heavily affected social activities. From a public survey, 88.1% of 903 participants reported that SARS strongly affected their daily lives along with undesirable impacts on social interactions with friends, family, and colleagues [10]. Moreover, the SARS outbreak had negative effects on healthcare workers and general publics, including financial loss, lifestyle changes, and masks interfering with social relationships [11,12]. Social isolation due to infection control intensely disrupts daily lives during a pandemic/epidemic. Based on past experiences with SARS and COVI-19, Taiwan established community partnerships among residents, health care professionals, and epidemic prevention experts to develop community capacity through planning and implementing effective biological disaster prevention policies [5].

In addition, other epidemics also had massively social impact. An epidemiological study investigating social-economic impact of pandemic influenza reported that 27% of families could not go to work and 18% of them suffered from economic burden due to school closure in Taiwan [13]. In another survey conducted in the U.S., schools were closed due to influenza B outbreak, and 24% of the households had missed work [14]. During the 2014–15 Ebola outbreak in West Africa, people encountered with socio-economic impacts, including reduced community cohesion, education loss, reduced child protection, and widespread job losses [15]. Several factors associated with community resilience were reported to be beneficial for publics recovering from Ebola outbreak, including level of knowledge, financial resources, and social capital (social support, social participation, and community bonds) [16].

### 1.3. Aim of Current Study

Because COVID-19 heavily interferes with social activities, a multi-dimensional assessment is warranted to assist authorities with developing or modifying pandemic policies to reduce negative impacts, appropriately mitigate the pandemic, and improve physical, social, and mental health. Considering the negative impact to mental health, implementing appropriate policies of social distancing and quarantine in rational manners is vitally important. To date, comprehensive studies remain insufficient in regards to evaluating the impact of COVID-19 on social activities and mental health. Given the paucity of relevant research and discourse, we developed the Societal Influences Survey Questionnaire (SISQ). The SISQ allows to evaluate the effect of COVID19 on individuals along several item categories, including social distancing, social anxiety, social desirability, social information and social adaptation. We also test the validity and reliability of the questionnaire to verify the quality and applicability. We aimed to develop a useful tool with sufficient quality and availability in order to evaluate various social impacts on people during a pandemic.

## 2. Materials and Methods

### 2.1. Participants

Participants were recruited through an online advertisement posted on social media platforms (Facebook and Line) from 08 April 2020 to 30 April 2020. Participants were also recruited through public health education activities at public area in Kaohsiung, such as a neighborhood assembly center or waiting area of the outpatient department. Individuals residing in Taiwan were eligible for this study if they agreed to fill in either online survey or paper-and-pencil questionnaire. Online questionnaires were developed though Google Forms. The announcement of the study is listed on the first page (Supplementary Figure S1). Participation in the online survey was voluntary. Survey responses were kept anonymous. Those who chose written surveys were given informed consent forms consistent with global ethical standards [17]. Research assistants explained the procedures of completing the questionnaires. The inclusion criteria of participants were those who agreed to fill in the online survey after reading the announcement and singed the inform consent for written questionnaires after explaining by researchers. Those who exhibited any cognitive impairment that prevented them from understanding the goal of the study or from completing the questionnaires were not included in the written test. Moreover, data of online survey was excluded when the missing value existed. The study was approved by the Human Research Ethics Committee (HREC) at National Cheng Kung University (NCKU HREC-E-109-066-2). No incentives were provided for participants other than gratitude and appreciation for their volunteer contributions.

### 2.2. Measures

#### 2.2.1. Societal Influences Survey Questionnaire

The Societal Influences Survey Questionnaire (SISQ) was constructed in reference to the Impact of Events Scales (IES) [18]. It was developed by Horowitz and colleagues and used to estimate current subjective distress, related to a specific event. These questionnaires were suitable to evaluate the anxiety, avoidance, and re-experience for survivors after traumatic events. We applied the IES to develop SISQ to assess the psychological impact, change of social habits, and lifestyle change for the general public during COVID-19 pandemic. For instance, the question “I feel anxious or fearful due to the pandemic” was developed in reference to cluster 1 (assessment of anxiety about a specific event) of IES. To ensure the face validity of SISQ, the expert meetings were held to review the translated items of questionnaires and remove irrelevant contents. Several experts and translators were invited to the meeting. For example, Dr. Frank Huang-Chih Chou, who is also one of the co-authors, had published several studies regarding the psychosocial impacts of massive disasters in Taiwan, such as earthquake, gas explosion, and COVID-19 pandemic [19,20,21]. Each item of SISQ was reviewed by experts to verify the face and content validity. In order to ensure that participants could entirely understand the meaning of questionnaires, several colleagues were invited to go through the SISQ and it was revised according to their comments.

The fifteen-item SISQ contained five categories of assessment, including social distance, social anxiety, social desirability, social information, and social adaptation. Social distance included questionnaires to estimate how participants reduced social interaction with others. Social anxiety stood for level of anxiety associated with COVID-19. Social desirability included questionnaires to estimate level of confidence against COVID-19 and adherence to government’s implementations. Social information represented the tendency of participants to acquire information about COVID-19. Social adaptation contained questionnaires about if participants were aware of progress of pandemic overseas. All of above factors were discussed and formulate in the expert meeting. It was composed of 4-point Likert scale, with scores ranging from 1 (never), 2 (seldom), 3 (occasional), and 4 (often). The details of 15 items were listed in Supplementary Table S1.

#### 2.2.2. Demographic Characteristics

Data was recorded with the participants’ age, educational level, marital status, gender, religion, and occupation. All of the demographic information was identified as categorical factors.

#### 2.2.3. Statistical Analysis

Descriptive analysis was used to summarize the variables, including gender, occupation, categories of age, marital status, religion, and level of education. The impact of COIVD-19 pandemic on multiple domains were determined by SISQ for participants. To test the reliability of the questionnaires, internal consistency was estimated by Cronbach’s α, where a value greater than 0.5 indicated moderate reliability [22]. Exploratory factor analysis (EFA) and confirmatory factor analysis (CFA) were performed to estimate the construct validity. The EFA was conducted with SPSS statistical software (IBM SPSS Statistics for Windows, Version 23.0, Armonk, NY, USA: IBM Corp). According to the assumption that the factors are correlated, principal axis factor analysis was performed with Varimax rotation. Then, the Kaiser–Mayer–Olkin (KMO) measure of sampling adequacy and Bartlett test were applied. The data was suitable for factor analysis if the KMO value was more than 0.60 and significant statistics (*p* < 0.05) was estimated from Bartlett test [23]. Total variance explained (%) and factor loadings were also estimated. The amount of variance indicates how well a relevant notion can be measured [24]. Regarding the field of social sciences, where information may be less precise, it is acceptable to set the threshold at 50 percent of the total variance [25]. Since the significant Kolmogorov-Smirnov test (*p* < 0.001) represented the non-normal distribution for the CFA subsample, maximum likelihood with Satorra-Bentler correction was used to determine if the model data fit with the item-factor structures.

The CFA was conducted using Amos statistical software (IBM Amos Statistics for Windows, Version 23.0, Armonk, NY, USA: IBM Corp). Composite reliability (CR) and average variance extracted (AVE) were applied to verify the convergent validity and discriminant validity. It was suggested that level of CR and AVE should reach 0.6 and 0.33 as satisfaction [26]. In order to test the adequacy of CFA model, multiple indices were applied to test the goodness of the model fit. Among each of indices, the values indicating acceptable model fit are as following: Chi-Square goodness-of-fit test (χ^2^/df < 5.0); Incremental Fit Index (IFI ≥ 0.95); Comparative Fit Index (CFI ≥ 0.95); Tucker-Lewis Index (TLI ≥ 0.95); Incremental Fit Index (IFI ≥ 0.95); Adjusted Goodness of Fit Index (AGFI ≥ 0.95); Goodness of Fit Index (GFI ≥ 0.95); Normed Fit Index (NFI ≥ 0.95); Stanrdized Root Mean Square Residual (SRMR ≤ 0.05); and Root-mean Square Error of Approximation (RMSEA < 0.08) [27,28,29].

In order to estimate the difference of total scores for five factors (social distance, social anxiety, social desirability, social information, and social adaptation), independent samples T-test and analysis of variance (ANOVA) were applied for categorical variables. Occupation and marital status were transformed into dichotomous variables (Unemployed or not; with partners (married and cohabitation) or not) for independent samples T-test. Gender difference was estimated with ANOVA. In addition, age and education level were transformed to continuous variables (age: 1 = under 20 years old to 7 = above 70 years old; education level: 1 = uneducated to 6 = master or doctor). Pearson’s correlation was applied to estimate the correlation with total scores of five factors and continuous variables.

## 3. Results

### 3.1. Summary of Demographic Analysis

Initially, 2240 subjects filled in the questionnaires either online or in written form. However, after excluding subjects who replied the forms with missing values (*n* = 328), the data of 1912 participants (1265 females, 652 males, and 5 transgender) were entered in the analysis. One thousand four hundred and seventy-seven (77.2%) of the participants were recruited from online survey, and the others (435; 22.8%) used paper-and-pencil questionnaire. The remaining summaries of characteristics for all participants are listed in Table 1.

### 3.2. Construct Validity

#### 3.2.1. Exploratory Factor Analysis

After testing with EFA, the KMO coefficient of sampling adequacy was 0.78 which lies within the acceptable range. Furthermore, the Bartlett’s Test of Sphericity, which assesses whether a matrix differs from the identity matrix, yielded significant results (*p* < 0.001). It demonstrated that the matrix did not resemble the identity matrix, and it also supported the presence of factors within the data.

Principal axis factor analysis was carried out with Varimax rotation to determine the factor solutions. Our result verified the proposed five-factor solution for all 15 items, and it explained 58.86% of the total variance, which reached acceptable range. In brief, the result of EFA demonstrated that the factors extracted from 15 items could be appropriately interpretative for all 15 items. The details of five factors (social distance, social anxiety, social desirability, social information, and social adaptation) and factor loadings of each item were listed in Table 2.

#### 3.2.2. Confirmatory Factor Analysis

After processing the CFA, the CR values of each factor were at 0.569 to 0.783, and the AVE for each factor distributed around 0.32 to 0.65. Most of the values were within required range, indicating acceptable convergent and discriminant validity (Tabachnick and Fidell, 2007). The CFA fit indices also indicated the adequacy of the model (RMSEA = 0.036; GFI = 0.981; χ^2^/df = 3.49; SRMR = 0.032). Overall, the current model showed an acceptable fit to the data. In short, the 15-items questionnaires were developed with good validity, and it ensured that the operational definition was comparable to conceptual definition. The remaining information of the CFA were listed at Table 3 and Table 4.

#### 3.2.3. Reliability Test

The overall internal consistency coefficient (Cronbach’s α) of the SISQ was 0.74, and the Cronbach’s α of each subscale were within 0.57 to 0.76, indicating adequate reliability. Other information was shown at Table 2.

#### 3.2.4. Comparison and Correlation between Variables

In comparison with employed participants through independent samples T-test, unemployed participants scored lower in social distance (*t* = 3.16; *p* = 0.002) and social adaptation (*t* = −2.40; *p* = 0.017); but higher in social anxiety (*t* = −5.35; *p* < 0.001). Compared with participants with partners, those without partners scored lower in social distance (*t* = −6.97; *p* < 0.001), social desirability (t = −2.87; *p* = 0.004), and social information (*t* = −5.04; *p* < 0.001). According to ANOVA with post hoc analysis, females scored higher than males in social distance, and the remaining variables revealed insignificant difference (Table 5). In addition, elder age was significantly correlated with higher scores of social distance (*r* = 0.22; *p* = 0.006), social desirability (*r* = 0.05; *p* = 0.026), and social information (*r* = 0.07; *p* = 0.003). Higher level of education was significantly correlated with higher scores of desirability (*r* = 0.11; *p* < 0.001), social information (*r* = 0.09; *p* < 0.001), and social adaptation (*r* = 0.13; *p* < 0.001). Among them, the effect sizes of significant correlations were low regarding the cutoff values of Pearson correlation.

## 4. Discussion

In the current study, we developed the SISQ and tested the reliability and validity. It accounted for 58.86% of the total variance, indicating the five subscales were statistically appropriate, including social distance, social anxiety, social desirability, social information, and social adaptation. The construct validity (CFA and EFA) and reliability (Cronbach’s alpha) supported the adequacy of the scale’s psychometric properties. Therefore, the SISQ was shown to be a brief and proper measurement for estimating the multiple-dimensional impact on social activities for people suffering from the pandemic.

Participants recruited in the current study demonstrated the higher proportion of female and high educational level. Previous online investigations for different topics of COVID-19 revealed similar findings [30,31,32,33]. Females had higher risk perception to enhance universal precautions in the hospital [34], and it was speculated that they might be more interested in information about uncertain threats than males, such as a novel infectious disease. Therefore, an online survey regarding COVID-19 may easily get attention from females. In addition, we supposed that individuals with higher educational level could get online information easier than those with lower educational level.

### 4.1. Impact of Social Anxiety, Social Information, and Social Desirability

When faced with uncertainty and threat due to the pandemic, people suffer from mental health problems, such as increased anxiety. The online data indicates high proportion of anxiety symptoms (35.1%) among the Chinese in China during the COVID-19 outbreak [35]. Another study [36] also indicated higher levels of anxiety were correlated with social isolation and quarantine during the SARS pandemic. This is not unexpected that identification of anxiety levels during a pandemic/epidemic is critically important to understand and address undesirable public impacts to mental and social health. A Taiwanese study demonstrates that excessive anxiety because of COVID-19 is associated with lower subjective psychological wellbeing [37]. As a result, necessary intervention for acute distress during COVID-19 pandemic helps to promote mental health among publics. Furthermore, it should be considered that post-traumatic stress disorder may emerge at the remission stage of COVID-19 for those experiencing massively psychological trauma, such as lost their beloved ones due to COVID-19.

Regarding the factor of social information, we supposed that it is a protective behavior for individuals to get information or watch news about COVID-19. Publics can make proper decision to cope with COVID-19 when they acquire sufficient information about infection control, updated status of spread, and policies announced from authorities. The significant association between receiving information about COVID-19 from more sources and greater confidence was found in healthcare workers [32]. Moreover, information overload indirectly enhances intention of self-isolation [38], which is a recommended behavior for public health. However, it should be noticed that excessive media exposure to crisis-related news elevated anxiety and stress responses among people during the COVID-19 outbreak [39]. In addition, we assessed the factor “social desirability” to estimate the confidence of individuals during pandemic. Previous cross-sectional study in China indicated that lower confidence against COVID-19 was associated with lower level of knowledge about COVID-19 [33], where it was supposed to be hazardous for public health. Hence, assessment on the level of social desirability may help us identify the impacts of COVID-19 and the associated risk factors.

### 4.2. Impact of Social Distance and Social Adaptation on Infectious Disease

Social distancing had been suggested by WHO in order to limit the transmission of COVID-19 [2]. Therefore, it is considered to be a well-adaptive behavior during the COVID-19 outbreak. However, the negative impact of social distance should not be ignored, and it contribute to undesirable effects on economy and mental health. Social distance is more likely to considered as stigma or social class, something with negative connotations [40]. Another study reported that social distancing at the workplace was effective for infection control but likely to be economically disruptive for productivity during influenza outbreak [41]. As a result, authorities should provide sufficient support for publics to minimize the undesirable effects of social distance. On the other hand, the factor “social adaptation” was developed to explore how subjects were awarded for prevention from traveling on themselves or others. A Hong Kong survey reported a high level of travel-avoidance and social-distancing for citizens during the COVID-19 outbreak [42]. Assessment of social adaptation is helpful for exploring its entire impact on social activities.

### 4.3. Difference between Categorical Variables and Coorelation between Continous Variables

Unemployed participants scored lower in factors of social distance and social adaptation; but higher in social anxiety. These findings indicated that unemployed subjects could not cope with threatens of COVID-19 properly but were more anxious about pandemic. Previous study found that unemployed subjects tended to have unhealthy dietary habits during COVID-19 pandemic [43]. It may echo with the current study, where unemployed subjects could not cope healthily in either diet habits or protective behaviors against COIVD-19. Furthermore, those with employment instability during the COVID-19 pandemic had significantly higher psychological distress [44]. Similar findings were found among participants without partners, indicating that they were less intended to keep social distance, acquire related information, and follow the direction of authorities for infection control. In Saudi, married individuals were more likely to comply with good practices during COVID-19 pandemic, such as avoid handshakes, wash hands constantly, and wear a mask [45]. Similar to above study, the current study further include cohabitation, and verify the protective effect for those with partners (married and cohabitation). Although difference between transgender and male or female was not identified, females scored higher than males in total scores of social distance. This finding suggests that females were more cautious to take protective behaviors. Previous studies also revealed that females had higher risk perception and more complied with strategies of infection control [34,46]. Although the coefficient value was low at 0.22, elder individuals potentially acted to keep social distance. It might result from the higher mortality rate for elderly infected with COVID-19 [47].

### 4.4. Limitation

The current study bears several limitations that need to be addressed. Firstly, one of the factors (social desirability) did not reach the required level of CR and AVE. However, the factor loading, internal consistency, and overall adequacy of the model could satisfy the requirement. Second, the relatively higher proportion of female and high educational level for the recruited participants may limit the generalizability. Third, the SISQ was developed in the acute stage of COVID-19. It may be necessary to be re-validated if this stool is going to apply in other stage of COVID-19, such as plateau or recurrent stage. Finally, the current study recruited participants only in Taiwan, which may limit the generalizability and applicability to other populations.

## 5. Conclusions

In the critical moment of COVID-19′s global outbreak, it is imperative to develop an evaluative tool about societal impacts that is reliable and valid while efforts are made to reduce possible risks of infection during this critical period. Through a questionnaire which garners the general public’s opinion, we can find strategies and a common ground through communication. This prevents societal misunderstandings about epidemic prevention work, its purpose, and the goal for active epidemic prevention actions. Therefore, in the current study, we developed the SISQ, which was verified as a valuable and reliable tool and could provide with important targets for further intervention. The SISQ is developed as a self-administrated questionnaire, which is convenient for research team to assess the impacts of COCID-19. It can also estimate different impacts for different stage of COVID-19, such as acutely spreading stage, plateau, or remission stage. With five categories of comprehensive assessment for influences on peoples, this study established a foundation for further research in social interaction, psychological impact, and lifestyle during infectious disease pandemic. The SISQ can also be applicable for other infectious disease through minor modification. In addition, we also identified several differences between demographic factors for the five factors of SISQ. Further studies are warranted to extend the applicability of SISQ.

## Figures and Tables

**Table 1:** Sociodemographic characteristics of participants (*n* = 1912).

Variable	*n*	%
Gender	-	-
Male	652	34.1
Female	1265	65.6
Transgender	5	0.3
Occupation	*n*	%
Unemployed	385	20.1
Policeman or fireman	25	1.3
Civil servant	137	7.2
Labor	696	36.4
Healthcare worker	182	9.5
Teacher	336	17.6
Student	137	7.2
Others	14	0.7
Age	*n*	%
Under 20 years old	65	3.4
20–29 years old	178	9.3
30–39 years old	341	17.8
40–49 years old	618	32.3
50–59 years old	507	26.5
60–69 years old	186	9.7
Above 70 years old	17	0.9
Marital status	*n*	%
Single	484	25.3
Married	1282	67.1
Divorced	108	5.6
Widowed	25	1.3
Cohabitation	13	0.7
Religion	*n*	%
Not religious	628	32.8
Religious	1284	67.2
Education	*n*	%
Uneducated	1	0.1
Primary school	9	0.5
Junior high school	51	2.7
Senior high school	254	13.3
College	984	51.5
Master or Doctor	613	32.1

**Table 2:** Exploratory factor analysis for COVID-19 Societal Influences Survey Questionnaire.

Factors/Items	EFA (Varimax Rotation)			Reliability	
	Sum of Squared Loading (Eigenvalue)	Variance Explained (%)	Cumulative Variance Explained (%)	Cronbach’s Alpha	Factor Loading
Social Distance	3.621	24.137	24.137	0.640	-
I avoid communication with or encountering strangers.	-	-	-	-	0.702
I avoid close or personal contact with family members and/or people I am close to	-	-	-	-	0.669
I avoid going out, especially if I should require public transport	-	-	-	-	0.647
I reduce eating out	-	-	-	-	0.690
Social Anxiety	1.687	11.245	35.382	0.633	-
I worry about the pandemic affecting my work	-	-	-	-	0.662
I feel anxious or fearful due to the pandemic	-	-	-	-	0.746
I am bothered by social distancing during this period of epidemic response	-	-	-	-	0.669
I am worried about COVID-19 and its impacts on our society, politics and economy	-	-	-	-	0.666
Social Desirability	1.293	8.623	44.005	0.565	-
I believe that self-health management is helpful in controlling the spread of COVID-19	-	-	-	-	0.660
I have faith in our current government’s epidemic response and risk management	-	-	-	-	0.751
I comply with the government’s implementations of epidemic response in the community	-	-	-	-	0.713
Social Information	1.231	8.209	52.213	0.756	-
I constantly check for latest pandemic news updates via television, computer or phone	-	-	-	-	0.881
I continuously seek out information regarding COVID-19.	-	-	-	-	0.794
Social Adaptation	0.997	6.644	58.858	0.659	-
I am more cautious of residents from severely impacted areas	-	-	-	-	0.782
I avoid or cancel traveling overseas	-	-	-	-	0.886

Kaiser_Meyer–Olkin (KMO) Measure of Sampling Adequacy: 0.783, Bartlett’s Test of Sphericity: <0.001, Overall Cronbach’s Alpha: 0.739.

**Table 3:** Confirmatory factor analysis for Coronavirus disease 2019 (COVID-19) Societal Influences Survey Questionnaire.

Factors/Items	Factor Loading	Square Multiple Correlation (SMC or *R*^2^)	Composite Reliability (CR)	Average Variance Extracted (AVE)
Social Distance	-	-	0.651	0.322
I avoid communication with or encountering strangers.	0.527	0.277	-	-
I avoid close or personal contact with family members and/or people I am close to	0.463	0.214	-	-
I avoid going out, especially if I should require public transport	0.662	0.438	-	-
I reduce eating out	0.599	0.359	-	-
Social Anxiety	-	-	0.659	0.339
I worry about the pandemic affecting my work	0.543	0.295	-	-
I feel anxious or fearful due to the pandemic	0.773	0.597	-	-
I am bothered by social distancing during this period of epidemic response	0.378	0.143	-	-
I am worried about COVID-19 and its impacts on our society, politics, and economy	0.565	0.319	-	-
Social Desirability	-	-	0.569	0.316
I believe that self-health management is helpful in controlling the spread of COVID-19	0.719	0.516	-	-
I have faith in our current government’s epidemic response and risk management	0.446	0.199	-	-
I comply with the government’s implementations of epidemic response in the community	0.482	0.232	-	-
Social Information	-	-	0.783	0.651
I constantly check for latest pandemic news updates via television, computer, or phone	0.649	0.421	-	-
I continuously seek out information regarding COVID-19	0.939	0.881	-	-
Social Adaptation	-	-	0.736	0.604
I am more cautious of residents from severely impacted areas	0.971	0.944	-	-
I avoid or cancel traveling overseas	0.515	0.265	-	-

**Table 4:** The indices of goodness-of-fit index for confirmatory factor analysis (CFA).

Goodness of Fit Index	Estimates	Acceptable Ranges
χ^2^/df	3.49	<5.0
RMESA	0.036	<0.08
GFI	0.981	>0.9
AGFI	0.972	>0.9
NFI	0.950	≥0.95
CFI	0.963	≥0.95
IFI	0.963	≥0.95
TLI	0.951	≥0.95
SRMR	0.032	<0.05

RMESA: Root-mean Square Error of Approximation; GFI: Goodness of Fit Index; AGFI: Adjusted Goodness of Fit Index; NFI: Normed Fit Index; CFI: Comparative Fit Index; IFI: Incremental Fit Index; TLI: Tucker-Lewis Index; SRMR: Standardized Root Mean Square Residual.

**Table 5:** Comparison of total scores for five scores across gender estimated with ANOVA.

Factor/Gender	Mean (SD)	Homogeneity of Variances	ANOVA Statistic (*p*-Value)	Post Hoc Analysis
Social Distance	-	<0.001 ^a^	16.91 (<0.001 ***) ^d^	Female > Male ^e^
Female	13.23 (2.38)	-	-	Male = Transgender (N.S.)
Male	12.37 (2.81)	-	-	Female = Transgender (N.S.)
Transgender	11.80 (3.35)	-	-	-
Social Anxiety	-	0.826 ^b^	0.19 (0.831) ^c^	N.S. ^f^
Female	10.58 (2.69)	-	-	-
Male	10.54 (2.74)	-	-	-
Transgender	11.20 (2.77)	-	-	-
Social Desirability	-	<0.001 ^a^	3.71 (0.077) ^d^	N.S. ^e^
Female	11.49 (1.02)	-	-	-
Male	11.28 (1.33)	-	-	-
Transgender	10.60 (2.07)	-	-	-
Social Information	-	0.003 ^a^	2.46 (0.159) ^d^	N.S. ^e^
Female	7.12 (1.22)	-	-	-
Male	6.96 (1.27)	-	-	-
Transgender	6.00 (2.35)	-	-	-
Social Adaptation	-	0.027 ^a^	1.94 (0.167) ^d^	N.S. ^e^
Female	7.03 (1.44)	-	-	-
Male	6.90 (1.55)	-	-	-
Transgender	7.20 (1.30)	-	-	-

^a^: The assumption of Homogeneity of variance for one-way ANOVA was violated (*p* < 0.05); ^b^: The assumption of Homogeneity of variance for one-way ANOVA was not violated (*p* ≥ 0.05); ^c^: F statistic was used when the assumption of Homogeneity of variance was not violated; ^d^: Brown-Forsythe statistic was used when the assumption of Homogeneity of variance was violated; ^e^: Post hoc analysis with Dunnett’s T3 test; ^f^: Post hoc analysis with Fisher’s Least Significant Difference (LSD) test; ***: statistic significant (*p* < 0.05); N.S.: non-significant (*p* ≥ 0.05).

## Supplementary Materials

The following are available online at https://www.mdpi.com/1660-4601/17/17/6246/s1, Figure S1: The cover page of the online questionnaire (Translated to English), Table S1: Societal Influences Survey Questionnaires (SISQ). Table S2: Descriptive statistics of Societal Influences Survey Questionnaires (SISQ).

## References

[1] Bartsch S.M., Ferguson M.C., McKinnell J.A., O’Shea K.J., Wedlock P.T., Siegmund S.S., Lee B.Y. (2020). The potential health care costs and resource use associated with COVID-19 in the United States. Health Aff..

[2] World Health Organization Coronavirus Disease (COVID-19) Advice for Public. https://www.who.int/emergencies/diseases/novel-coronavirus-2019/advice-for-public.

[3] Chen N., Zhou M., Dong X., Qu J., Gong F., Han Y., Qiu Y., Wang J., Liu Y., Wei Y. (2020). Epidemiological and clinical characteristics of 99 cases of 2019 novel coronavirus pneumonia in Wuhan, China: A descriptive study. Lancet.

[4] Coibion O., Gorodnichenko Y., Weber M. (2020). Labor Markets During the COVID-19 Crisis: A Preliminary View.

[5] Lo H.A., Huang J.J., Chen C.C., Tsai D., Chou F.H., Shieh V. (2020). Community-based epidemic prevention in Taiwan: Combating the coronavirus disease—2019 crisis. Disaster Med. Public Health Prep..

[6] Alkhamees A.A., Alrashed S.A., Alzunaydi A.A., Almohimeed A.S., Aljohani M.S. (2020). The psychological impact of COVID-19 pandemic on the general population of Saudi Arabia. Compr. Psychiatry.

[7] Banna M.H.A., Sayeed A., Kundu S., Christopher E., Hasan M.T., Begum M.R., Kormoker T., Dola S.T.I., Hassan M.M., Chowdhury S. (2020). The impact of the COVID-19 pandemic on the mental health of the adult population in Bangladesh: A nationwide cross-sectional study. Int. J. Env. Health Res..

[8] Cheng S.Y., Wang C.J., Shen A.C., Chang S.C. (2020). How to safely reopen colleges and universities during COVID-19: Experiences from Taiwan. Ann. Intern. Med..

[9] Venkatesh A., Edirappuli S. (2020). Social distancing in covid-19: What are the mental health implications?. BMJ.

[10] Lee S., Chan L.Y., Chau A.M., Kwok K.P., Kleinman A. (2005). The experience of SARS-related stigma at Amoy Gardens. Soc. Sci. Med..

[11] Straus S.E., Wilson K., Rambaldini G., Rath D., Lin Y., Gold W.L., Kapral M.K. (2004). Severe acute respiratory syndrome and its impact on professionalism: Qualitative study of physicians’ behaviour during an emerging healthcare crisis. BMJ.

[12] Svoboda T., Henry B., Shulman L., Kennedy E., Rea E., Ng W., Wallington T., Yaffe B., Gournis E., Vicencio E. (2004). Public health measures to control the spread of the severe acute respiratory syndrome during the outbreak in Toronto. N. Engl. J. Med..

[13] Chen W.C., Huang A.S., Chuang J.H., Chiu C.C., Kuo H.S. (2011). Social and economic impact of school closure resulting from pandemic influenza A/H1N1. J. Infect..

[14] Johnson A.J., Moore Z.S., Edelson P.J., Kinnane L., Davies M., Shay D.K., Balish A., McCarron M., Blanton L., Finelli L. (2008). Household responses to school closure resulting from outbreak of influenza B, North Carolina. Emerg. Infect. Dis..

[15] Elston J.W., Cartwright C., Ndumbi P., Wright J. (2017). The health impact of the 2014–15 Ebola outbreak. Public Health.

[16] Bhandari S., Alonge O. (2020). Measuring the resilience of health systems in low- and middle-income countries: A focus on community resilience. Health Res. Policy Syst..

[17] Department of Hegalth, Education, and Welfare, National Commission for the Protection of Human Subjects of Biomedical and Behavioral Research (2014). The Belmont Report. Ethical principles and guidelines for the protection of human subjects of research. J. Am. Coll. Dent..

[18] Horowitz M., Wilner N., Alvarez W. (1979). Impact of event scale: A measure of subjective stress. Psychosom. Med..

[19] Hsu S.T., Chou L.S., Chou F.H., Hsieh K.Y., Chen C.L., Lu W.C., Kao W.T., Li D.J., Huang J.J., Chen W.J. (2020). Challenge and strategies of infection control in psychiatric hospitals during biological disasters—From SARS to COVID-19 in Taiwan. Asian J. Psychiatry.

[20] Huang J.J., Wu T.G., Chen Y.C., Chiu J.Y., Chou P., Chou F.H. (2018). A preliminary report on psychiatric impairments and quality of life among Kaohsiung gas explosion victims 6 months after the event. Qual. Life Res..

[21] Tsai K.Y., Chou P., Chou F.H., Su T.T., Lin S.C., Lu M.K., Ou-Yang W.C., Su C.Y., Chao S.S., Huang M.W. (2007). Three-year follow-up study of the relationship between posttraumatic stress symptoms and quality of life among earthquake survivors in Yu-Chi, Taiwan. J. Psychiatr. Res..

[22] Hinton P.R., McMurray I., Brownlow C. (2014). SPSS Explained.

[23] Tabachnick B.G., Fidell L.S. (2007). Using Multivariate Statistics.

[24] Hardy M.B.A. (2004). Handbook of Data Analysis.

[25] Beavers A.S., Lounsbury J.W., Richards J.K., Huck S.W., Skolits G., Esquivel S.L. (2013). Practical considerations for using exploratory factor analysis in educational research. Pract. Assess. Res. Eval..

[26] Fornell C., Larcker D.F. (1981). Evaluating structural equation models with unobservable variables and measurement error. J. Mark. Res..

[27] Fan X., Sivo S.A. (2005). Sensitivity of Fit Indexes to misspecified structural or measurement model components: Rationale of two-index strategy revisited. Struct. Equ. Model. Multidiscip. J..

[28] Hu L.T., Bentler P.M. (1999). Cutoff criteria for fit indexes in covariance structure analysis: Conventional criteria versus new alternatives. Struct. Equ. Model. Multidiscip. J..

[29] Kline T. (2005). Psychological Testing: A Practical Approach to Design and Evaluation.

[30] Geldsetzer P. (2020). Use of rapid online surveys to assess people’s perceptions during infectious disease outbreaks: A Cross-sectional survey on COVID-19. J. Med. Internet Res..

[31] Li D.J., Ko N.Y., Chen Y.L., Wang P.W., Chang Y.P., Yen C.F., Lu W.H. (2020). COVID-19-related factors associated with sleep disturbance and suicidal thoughts among the Taiwanese public: A Facebook survey. Int. J. Environ. Res. Public Health.

[32] Wang P.W., Lu W.H., Ko N.Y., Chen Y.L., Li D.J., Chang Y.P., Yen C.F. (2020). COVID-19-related information sources and the relationship with confidence in people coping with COVID-19: Facebook survey study in Taiwan. J. Med. Internet Res..

[33] Zhong B.L., Luo W., Li H.M., Zhang Q.Q., Liu X.G., Li W.T., Li Y. (2020). Knowledge, attitudes, and practices towards COVID-19 among Chinese residents during the rapid rise period of the COVID-19 outbreak: A quick online cross-sectional survey. Int. J. Biol. Sci..

[34] Gershon R.R., Vlahov D., Felknor S.A., Vesley D., Johnson P.C., Delclos G.L., Murphy L.R. (1995). Compliance with universal precautions among health care workers at three regional hospitals. Am. J. Infect. Control.

[35] Huang Y., Zhao N. (2020). Mental health burden for the public affected by the COVID-19 outbreak in China: Who will be the high-risk group?. Psychol. Health Med..

[36] Hawryluck L., Gold W.L., Robinson S., Pogorski S., Galea S., Styra R. (2004). SARS control and psychological effects of quarantine, Toronto, Canada. Emerg. Infect. Dis..

[37] Ko N.Y., Lu W.H., Chen Y.L., Li D.J., Wang P.W., Hsu S.T., Chen C.C., Lin Y.H., Chang Y.P., Yen C.F. (2020). COVID-19-related information sources and psychological well-being: An online survey study in Taiwan. Brain Behav. Immun..

[38] Farooq A., Laato S., Islam A. (2020). Impact of online information on self-isolation intention during the COVID-19 pandemic: Cross-sectional study. J. Med. Internet Res..

[39] Garfin D.R., Silver R.C., Holman E.A. (2020). The novel coronavirus (COVID-2019) outbreak: Amplification of public health consequences by media exposure. Health Psychol..

[40] O’Brien A. (2020). Covid 19: Transcending social distance. J. Psychiatr. Ment. Health Nurs..

[41] Rashid H., Ridda I., King C., Begun M., Tekin H., Wood J.G., Booy R. (2015). Evidence compendium and advice on social distancing and other related measures for response to an influenza pandemic. Paediatr. Respir. Rev..

[42] Kwok K.O., Li K.K., Chan H.H.H., Yi Y.Y., Tang A., Wei W.I., Wong S.Y.S. (2020). Community Responses during Early Phase of COVID-19 Epidemic, Hong Kong. Emerg. Infect. Dis..

[43] Gornicka M., Drywien M.E., Zielinska M.A., Hamulka J. (2020). Dietary and lifestyle changes during COVID-19 and the subsequent lockdowns among Polish adults: A Cross-sectional online survey PLifeCOVID-19 study. Nutrients.

[44] Mimoun E., Ben Ari A., Margalit D. (2020). Psychological aspects of employment instability during the COVID-19 pandemic. Psychol. Trauma.

[45] Almutairi A.F., BaniMustafa A., Alessa Y.M., Almutairi S.B., Almaleh Y. (2020). Public trust and compliance with the precautionary measures against COVID-19 employed by authorities in Saudi Arabia. Risk Manag. Healthc. Policy.

[46] Ward D. (2004). Gender differences in compliance with infection control precautions. Br. J. Infect. Control.

[47] Zhou F., Yu T., Du R., Fan G., Liu Y., Liu Z., Xiang J., Wang Y., Song B., Gu X. (2020). Clinical course and risk factors for mortality of adult inpatients with COVID-19 in Wuhan, China: A retrospective cohort study. Lancet.

