# Supplementary material for: Development and Implementation of Societal Influences Survey Questionnaire (SISQ) for Peoples during COVID-19 Pandemic: A Validity and Reliability Analysis
Source: Int J Environ Res Public Health. 2020 Aug 27;17(17):6246. doi: 10.3390/ijerph17176246 (PMC7503324; doi:10.3390/ijerph17176246)
Supplement: Supplementary file 1 [file ijerph-17-06246-s001.pdf]

Supplementary Figure 1.

The cover page of the online questionnaire (Translated to English)

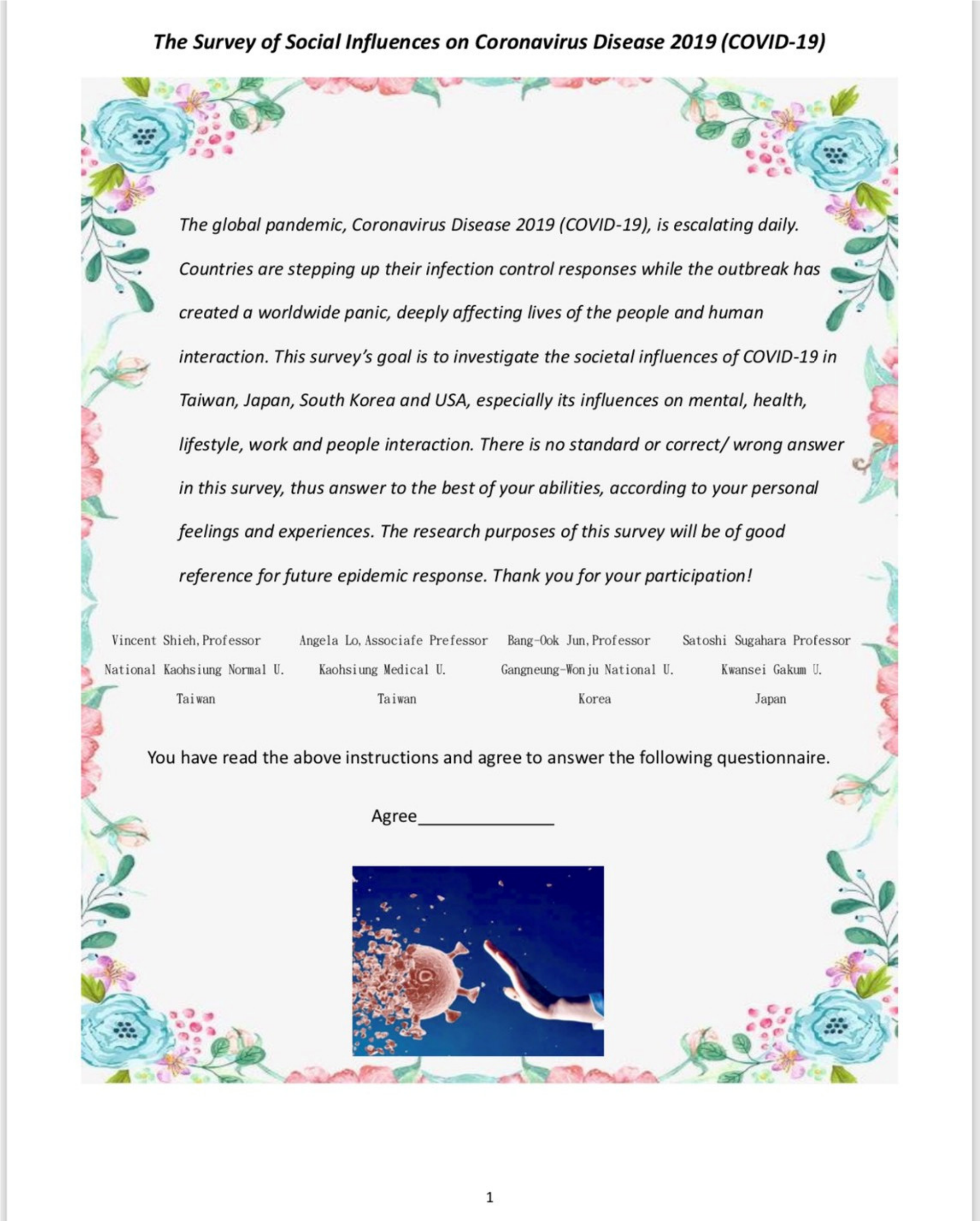

The Survey of Social Influences on Coronavirus Disease 2019 (COVID-19)

The global pandemic, Coronavirus Disease 2019 (COVID-19), is escalating daily. Countries are stepping up their infection control responses while the outbreak has created a worldwide panic, deeply affecting lives of the people and human interaction. This survey's goal is to investigate the societal influences of COVID-19 in Taiwan, Japan, South Korea and USA, especially its influences on mental, health, lifestyle, work and people interaction. There is no standard or correct/ wrong answer in this survey, thus answer to the best of your abilities, according to your personal feelings and experiences. The research purposes of this survey will be of good reference for future epidemic response. Thank you for your participation!

Vincent Shieh, Professor	Angela Lo, Associafe Prefessor	Bang-Ook Jun, Professor	Satoshi Sugahara Professor
National Kaohsiung Normal U.	Kaohsiung Medical U.	Gangneung-Wonju National U.	Kwansei Gakum U.
Taiwan	Taiwan	Korea	Japan

You have read the above instructions and agree to answer the following questionnaire.

Agree______________

Supplementary Table. 1. Societal Influences Survey Questionnaires (SISQ).

	How has COVID-19 impacted your society?	*Never 0%*	*Rarely 10%*	*Sometimes 30%*	*Often 60%*
**1**	I constantly check for latest pandemic news updates via television, computer or phone	□	□	□	□
2	I avoid communication with or encountering strangers.	□	□	□	□
3	I avoid close or personal contact with family members and/or people I am close to	□	□	□	□
4	I worry about the pandemic affecting my work	□	□	□	□
5	I feel anxious or fearful due to the pandemic	□	□	□	□
6	I am more cautious of residents from severely impacted areas	□	□	□	□
7	I avoid or cancel traveling overseas<br>(Answer "60%", if you have cancelled an overseas trip. Answer "10%" or "30%", if you are still considering your cancellation.)	□	□	□	□
8	I avoid going out, especially if I should require public transport	□	□	□	□
9	I continuously seek out information regarding COVID-19.	□	□	□	□
10	I believe that self-health management is helpful in controlling the spread of COVID-19	□	□	□	□
11	I have faith in our current government's epidemic response and risk management	□	□	□	□
12	I am bothered by social distancing during this period of epidemic response	□	□	□	□
13	I comply with the government's implementations of epidemic response in the community	□	□	□	□
14	I am worried about COVID-19 and its impacts on our society, politics and economy	□	□	□	□
15	I reduce eating out	□	□	□	□

Supplementary Table. 2. Descriptive statistics of Societal Influences Survey Questionnaires (SISQ).

**Factors/Items**	**Means**	**Standard Deviation**
Social Distance	-	-
I avoid communication with or encountering strangers.	3.59	0.85
I avoid close or personal contact with family members and/or people I am close to	2.85	1.14
I avoid going out, especially if I should require public transport	3.46	0.86
I reduce eating out	3.05	0.98
Social Anxiety	-	-
I worry about the pandemic affecting my work	2.56	1.22
I feel anxious or fearful due to the pandemic	2.41	0.93
I am bothered by social distancing during this period of epidemic response	2.28	1.00
I am worried about COVID-19 and its impacts on our society, politics and economy	3.32	0.77
Social Desirability	-	-
I believe that self-health management is helpful in controlling the spread of COVID-19	3.82	0.50
I have faith in our current government's epidemic response and risk management	3.71	0.58
I comply with the government's implementations of epidemic response in the community	3.88	0.48
Social Information	-	-
I constantly check for latest pandemic news updates via television, computer or phone	3.50	0.72
I continuously seek out information regarding COVID-19.	3.57	0.67
Social Adaptation	-	-
I am more cautious of residents from severely impacted areas	3.54	1.06
I avoid or cancel traveling overseas	3.48	1.28
